# Melatonin Decreases Pulmonary Vascular Remodeling and Oxygen Sensitivity in Pulmonary Hypertensive Newborn Lambs

**DOI:** 10.3389/fphys.2018.00185

**Published:** 2018-03-06

**Authors:** Cristian R. Astorga, Alejandro González-Candia, Alejandro A. Candia, Esteban G. Figueroa, Daniel Cañas, Germán Ebensperger, Roberto V. Reyes, Aníbal J. Llanos, Emilio A. Herrera

**Affiliations:** ^1^Laboratory of Vascular Function & Reactivity, Pathophysiology Program, ICBM, Faculty of Medicine, Universidad de Chile, Santiago, Chile; ^2^Department for the Woman and Newborn Health Promotion, Universidad de Chile, Santiago, Chile; ^3^Department of Mechanical Engineering, Faculty of Engineering, Universidad de Santiago de Chile, Santiago, Chile; ^4^Perinatal Physiology and Pathophysiology Unit, Pathophysiology Program, ICBM, Faculty of Medicine, Universidad de Chile, Santiago, Chile; ^5^International Center for Andean Studies, Universidad de Chile, Santiago, Chile

**Keywords:** neonatal pulmonary hypertension, vascular remodeling, hypoxic pulmonary vasoconstriction, chronic hypoxia, oxidative stress, melatonin

## Abstract

**Background:** Chronic hypoxia and oxidative stress during gestation lead to pulmonary hypertension of the neonate (PHN), a condition characterized by abnormal pulmonary arterial reactivity and remodeling. Melatonin has strong antioxidant properties and improves pulmonary vascular function. Here, we aimed to study the effects of melatonin on the function and structure of pulmonary arteries from PHN lambs.

**Methods:** Twelve lambs (*Ovis aries*) gestated and born at highlands (3,600 m) were instrumented with systemic and pulmonary catheters. Six of them were assigned to the control group (CN, oral vehicle) and 6 were treated with melatonin (MN, 1 mg.kg^−1^.d^−1^) during 10 days. At the end of treatment, we performed a graded oxygenation protocol to assess cardiopulmonary responses to inspired oxygen variations. Further, we obtained lung and pulmonary trunk samples for histology, molecular biology, and immunohistochemistry determinations.

**Results:** Melatonin reduced the *in vivo* pulmonary pressor response to oxygenation changes. In addition, melatonin decreased cellular density of the media and diminished the proliferation marker KI67 in resistance vessels and pulmonary trunk (*p* < 0.05). This was associated with a decreased in the remodeling markers α-actin (CN 1.28 ± 0.18 vs. MN 0.77 ± 0.04, *p* < 0.05) and smoothelin-B (CN 2.13 ± 0.31 vs. MN 0.88 ± 0.27, *p* < 0.05). Further, melatonin increased vascular density by 134% and vascular luminal surface by 173% (*p* < 0.05). Finally, melatonin decreased nitrotyrosine, an oxidative stress marker, in small pulmonary vessels (CN 5.12 ± 0.84 vs. MN 1.14 ± 0.34, *p* < 0.05).

**Conclusion:** Postnatal administration of melatonin blunts the cardiopulmonary response to hypoxia, reduces the pathological vascular remodeling, and increases angiogenesis in pulmonary hypertensive neonatal lambs.These effects improve the pulmonary vascular structure and function in the neonatal period under chronic hypoxia.

## Introduction

Pulmonary hypertension of the neonate (PHN) is a syndrome characterized by a failure in the mechanisms that decrease pulmonary vascular resistance (PVR) and pulmonary arterial pressure (PAP) after birth (Gersony et al., 1969; Lakshminrusimha et al., 2016). In lowland population, less than 1% of the neonates have respiratory distress syndrome (Keyes et al., 2003), whereas in highland population (>2,500 masl), this condition can reach up to 10% which includes PHN (Keyes et al., 2003; Peñaloza and Arias-Stella, 2007; Peñaloza, 2012). Further, hypoxia-induced PHN has been associated with pulmonary endothelial dysfunction, increased vascular reactivity, and remodeling (Herrera et al., 2007, 2008, 2010; Llanos et al., 2011; Torres et al., 2015). The vascular response known as hypoxic pulmonary vasoconstriction (HPV), is a rapid and reversible increase in the resistance of pulmonary arterioles in response to regional decrease of oxygen, a unique characteristic of the pulmonary circulation that optimizes the matching between ventilation and perfusion (West, 2016). However, in hypobaric environments such as the *Andean Altiplano*, the entire lungs become hypoxic and therefore increases PVR and right ventricular afterload (Herrera et al., 2007; Peñaloza and Arias-Stella, 2007). In fact, PHN denotes a vasoactive imbalance toward a vasoconstrictor tone which determines increased PVR at any oxygenation level (Herrera et al., 2007, 2008; Papamatheakis et al., 2013).

Conversely, vascular remodeling is considered as an active process where vascular cells and layers are affected, involving cell growth, cell death, cell migration, and extracellular matrix dynamics (Papamatheakis et al., 2013). These, processes are dependent on interactions between locally generated growth factors, vasoactive substances, and hemodynamic stimuli (Gibbons and Dzau, 1994). In PHN, the pulmonary vascular bed is characterized by an endothelial dysfunction, excessive muscularization of the pulmonary arterioles, and underdevelopment of the circulation (Murphy et al., 1981; Haworth and Hislop, 1982; Allen and Haworth, 1986; Ohara et al., 1991; Mulvany, 1999; Herrera et al., 2008; Gao and Raj, 2010; Papamatheakis et al., 2013). At birth, there are several critical changes in the structure and function of smooth muscle cells (SMCs), particularly in the medial layer of resistance arteries (Stenmark et al., 1988; Wohrley et al., 1995). For instance, the fetal SMCs have a synthetic (proliferative) phenotype with low contractile proteins expression, such as α-actin, myosin heavy chain (MHC) and smoothelin-B defining a rhomboidal-shaped cells overlaid arrangement (Rensen et al., 2007). These characteristics disappear after birth in healthy neonates; however, in PHN the SMCs conserve a high proliferation pattern leading to lack of the vascular wall thinning (Allen and Harworth, 1988; Gao and Raj, 2010) and therefore to an increased medial layer thickness and a decreased luminal area of the pulmonary arteries (Stenmark et al., 1987; Herrera et al., 2008). This increase in the medial:lumen ratio is a main hallmark of an inward remodeling of resistance arteries (Mulvany, 1999). Further, similar changes have been observed in large pulmonary arteries in newborns exposed to chronic hypoxia (Meyrick and Reid, 1982). The main pulmonary artery has 3 phenotypically distinct SMC subpopulations, organized in three zones or layers (L1, L2, and L3) (Frid et al., 1994, 1997a,b). The phenotype, growth, and matrix-producing capabilities are specific for of each of these zones, suggesting that medial SMC subpopulations exhibit differential proliferative responses to hypoxia (Frid et al., 1997b).

PHN is characterized by a low angiogenic capacity of endothelial cells and an increased alveolar/arterial ratio (Mahajan et al., 2015). Interestingly, the vascular endothelial growth factor (VEGF), as the main angiogenic mediator, is markedly increased under chronic hypoxia (Christou et al., 1998; Nadeau et al., 2005). However, little is known about VEGF response in PHN due to developmental chronic hypoxia, and how HIF-1α regulate VEGF expression under these conditions.

Further, it has been shown that hypoxia and oxidative stress play an important role in the development of the structural alterations associated with PHN (Herrera et al., 2007, 2015; Torres et al., 2015). In physiological conditions, reactive oxygen species (ROS) may act as modulators for a wide range of cellular mechanisms (Dröge, 2002; Konduri et al., 2007; Murphy et al., 2011). However, ROS increase, for instance during hypoxia, can determine oxidative stress (Clanton, 2007; Sylvester et al., 2012), a condition that increases the expression and/or activity of multiple growth factors involved in vascular function, cell growth and proliferation (Dröge, 2002; Hartney et al., 2011; Aggarwal et al., 2013). In fact, ROS induce the mitogen-activated protein kinase (MAPK), which is part of a common signaling pathway that induces cell division. Conversely, ROS increase the expression of cyclins that modulate the function cyclin-dependent kinases (CDKs), activating growth factors associated with the control of the cell cycle (Wedgwood et al., 2013; Wedgwood and Steinhorn, 2014). Further, transcription factors that induce pathological remodeling and vasoconstrictor processes such as HIF-1 are stabilized by ROS and by mechanical stretching observed in the pulmonary circulation of PHN at high altitude (Farías et al., 2016; Veith et al., 2016).

Currently, the treatment for PHN considers timely and precise interventions such as inhaled NO, controlled oxygen administration, PDE5 inhibitors and even extracorporeal membrane oxygenation (Lakshminrusimha et al., 2016). However, these therapeutic strategies do not markedly reduce the mortality and the long-term outcomes remain poor for children with PHN (Lakshminrusimha et al., 2016). One of the possible explanations of the partial failure of these treatments is that they target mostly vasodilation but none of them are significant antioxidants or anti-remodeling agents. Considering the detrimental effects of oxidative stress during perinatal life, several antioxidant therapies have been proposed (Perrone et al., 2010; Miller et al., 2012), but few have been specifically tested for PHN (Torres et al., 2015).

Melatonin is a neurohormone with a number of benefits on vascular function and antioxidant properties (Rodriguez et al., 2004; Hardeland, 2005; Torres et al., 2015). Melatonin antioxidant attributes are dependent on at least three properties, being a direct scavenger of ROS, its ability to upregulate antioxidant enzymes (superoxide dismutase, catalase and glutathione peroxidase) and to negatively modulate pro-oxidant activity (Rodriguez et al., 2004; Hardeland, 2005). Recently, we demonstrated that melatonin has a mild pulmonary vasodilator effect and markedly reduces oxidative stress and improves *ex vivo* vascular function in neonates with PHN (Torres et al., 2015). Therefore, in this study we tested the hypothesis that a postnatal melatonin treatment has vasodilator and anti-remodeling effects on the pulmonary circulation of neonatal lambs with PHN.

## Materials and methods

All animal care, procedures and experimentation were approved by the Ethics Committee of the Faculty of Medicine, University of Chile (CBA # 0398 FMUCH) and the Advisory Committee on Bioethics of FONDECYT (N°018/FONDECYT/Medicine/0097), and they were carried out according to international standards following the Guide for the Care and Use of Laboratory Animals published by the US National Institutes of Health (NIH Publication No. 85-23, revised 1996).

### Animals

Twelve newborn sheep (*Ovis aries*) were gestated, born and studied at high altitude (INCAS Research Station, Putre, 3,600 m), and randomly divided in two groups. These animals develop PHN due to their development under chronic hypoxia at high altitude (Herrera et al., 2007, 2015; Torres et al., 2015). The control group received vehicle (CN, *n* = 6, 1.4% ethanol 0.5 mL kg^−1^) and the treated group received a dose of melatonin (MN, *n* = 6, Melatonin 1 mg kg^−1^ in 1.4% ethanol 0.5 mL kg^−1^) in a daily fashion during 10 days (3–12 days old). Both treatments were given orally, at dusk (20:00 h) to follow melatonin circadian rhythm and avoid chronodisruption.

### *In vivo* experiments

All lambs were instrumented at 3 days old for daily hemodynamic and blood gases monitoring as described previously (Herrera et al., 2007, 2008). In brief, lambs were anesthetized with a ketamine–xylazine association (10/0.04 mg/kg I.M.) with additional local infiltration of 2% lidocaine in the incision area. A polyvinyl catheter was placed in the descending aorta and a Swan–Ganz catheter was placed in the pulmonary artery (Herrera et al., 2007, 2008). After 10 days of treatment, we performed a graded oxygenation protocol, in which the FiO_2_ was modified to achieve arterial PO_2_ between 30 and 120 mmHg in a 5–10 mmHg stepwise manner (Herrera et al., 2007). This experimental protocol includes about 12 steps and last for 75 min, where PAP, systemic arterial pressure (SAP) and heart rate (HR) were recorded continuously (Herrera et al., 2007). Further, cardiac output was calculated at the end of each oxygenation step. All the cardiovascular variables were plotted and correlated with arterial PO_2_. All *in vivo* measurements were performed in un-anesthetized and awaked animals, between 9:00 and 12:00 of the day.

The day after the graded oxygenation (12–13 days old), lambs underwent euthanasia with an overdose of sodium thiopentone (100 mg kg^−1^, slow I.V. infusion) for tissue sampling.

### Pulmonary morphostructural analyses

At post-mortem, the left lung was extracted and perfused at 25 mmHg via pulmonary artery with saline for blood removal and 4% paraformaldehyde (PFA) for fixation. Afterwards, 1 cm^3^ pulmonary blocks and the main pulmonary artery were immersed-fixed with 4% PFA for 24 h at 4°C, followed by conservation in PBS + sodium azide 0.01% at 4°C. Fixed samples were embedded in paraffin and cut in 4 μm slides. Hematoxilin-Eosin and Van Gieson stainings were performed for vascular morphometry. Images were captured at 10x and 40x with a microscope (Olympus BX-41) coupled to a digital camera and computer. The analysis of the microphotographs was performed with the software Image Pro-Plus 6.2 (Media Cybernetics, Inc., Rockville, MD, USA). Briefly, luminal, medial and adventitial perimeters were measured for the estimation of the internal and external diameters. We further calculate luminal, wall, medial, and adventitial areas; luminal/vascular area and luminal/wall area ratios. Finally, media cellular density was determined for each selected artery (Herrera et al., 2008). Twenty to twenty-five representative resistance pulmonary arteries from each animal were selected for these analyses. Further, vascular density (number of arteries/area) and % luminal area (Σ luminal area/total area) in lung samples were determined for resistance arteries (50–200 μm of internal diameter).

The analysis of the main pulmonary trunk morphostructure consisted in the determination of the internal diameter (lumen), the external diameter (external adventitia) and the tunica media thickness. Moreover, the cell density and thickness of L1, L2, and L3 zones of the tunica media were determined as previously described (Frid et al., 1994). Briefly, we separated the 3 medial layers as the subendothelial layer (termed here L1), an intermediate-sized middle layer (termed L2), and a thick outer layer (termed L3). Previous studies have shown that these layers have specific patterns of cell arrangement and elastic lamellar distribution (Frid et al., 1994, 1997a,b).

### Pulmonary immunohistochemistry

Proliferation in the tunica media was assessed by immunohistochemistry using the anti-Ki67 monoclonal antibody (MAB3242, Millipore, Merck). Further, immunolocalization of VEGF-A in the vascular wall was studied using the anti-VEGF-A monoclonal antibody (MAI-16626, Pierce Biotechnology). In addition, immunolocalization of nitrotyrosine in lung tissue was evaluated by the anti-nitrotyrosine monoclonal antibody (Clone 1A6, Millipore, Merck). Briefly, the tissue sections were exposed to retrieval buffer 1X for antigen retrieval (Target Retrieval Solution, Dako) at 120°C for 25 min. The primary antibodies anti-Ki67 and VEGF-A were incubated in bovine serum albumin 1% (1:100) for 3 h and then the slides were incubated with an anti-mouse polymer (EnVision System-HRP, Dako) for 1 extra hour. Finally, the immunoreaction was revealed with diaminobencidine and nuclear stain was performed with Harris hematoxylin. Twenty to twenty-five representative resistance pulmonary arteries (50–200 μm of internal diameter) from each animal were photographed and the percentage of proliferative cells were determined by counting the positive and total cells.

### Protein expression

Protein expression of α-actin, MHC, smoothelin-B, VEGF-A, HIF-1α and β-actin was determined by immunoblot in total lung lysates with specific primary antibodies (A-5228 anti-α-actin, Sigma-Aldrich; anti-MHC MAB3572, Millipore; MAB 3242, anti-Smoothelin-B, Millipore; MAI-16626 anti-VEGF-A, Pierce Biotechnology; sc-10790 anti-HIF-1α, Santa Cruz Biotechnology, and MA1-91399 anti-β-actin (AC-15), Thermo Fisher Scientific) as described elsewhere (Herrera et al., 2008; Torres et al., 2015). Appropriate secondary antibodies were used and signals detected by chemoluminiscence. The immunoblot photographs were obtained with a scanner (Odyssey Imaging System, Li-Cor Biosciences) and quantified by densitometric analysis (Image J, NIH).

### Statistical analyses

All data were expressed as means ± SEM. Shapiro-Wilk test was used to assess normality of the data. Ratios and percentages were arcsine-transformed prior to statistical analysis. The graded oxygenation hemodynamic responses were fitted using a non-linear regression function (SAP, PAP, HR, CO). Inflection point of the curves were calculated directly from the quadratic polynomials. For this, we obtained an initial and a final slope (line). A first estimate of the slope is made by selecting the points in one side of the curve until a 5% variation of the slope value is reached. This is made in order to obtain a representative value of the slope with as many points as possible and it was applied to each side of the curve. The inflection point represents the intersection of both slopes (Bustos et al., 2017). All results were compared statistically by Mann-Whitney test unless otherwise stated. Significant differences were accepted when *P* ≤ 0.05 (Prism 5.0; GraphPad).

## Results

### Graded oxygenation

During basal conditions, arterial and pulmonary blood gases were similar between both groups (Table 1). During the graded oxygenation experiment, PaO_2_, SaO_2_, O_2_ content, and PCO_2_ changed during each step, with no changes in pH or Hb concentration (data not shown).

**Table 1:** Basal arterial blood gases.

	**Control (CN)**	**Melatonin (MN)**
pH	7.473 ± 0.012	7.479 ± 0.010
PCO_2_, mmHg	36.3 ± 1.8	35.1 ± 1.2
PO_2_, mmHg	45.1 ± 1.4	46.8 ± 0.8
Hb, g.dL^−1^	12.88 ± 0.62	12.84 ± 0.40
SaO_2_, %	71.4 ± 1.6	72.9 ± 3.6
O_2_ cont, mL.dL^−1^	12.12 ± 0.64	12.46 ± 0.41

*Arterial pH, PCO_2_, PO_2_, hemoglobin concentration (Hb), Hemoglobin saturation by oxygen (SaO_2_), and arterial blood oxygen content (O_2_ cont) in neonatal sheep during the basal period of the graded oxygenation protocol. Groups are control (CN, n = 6) and melatonin treated (MN, n = 6) lambs. Values are means ± SEM*.

**Figure 1:**
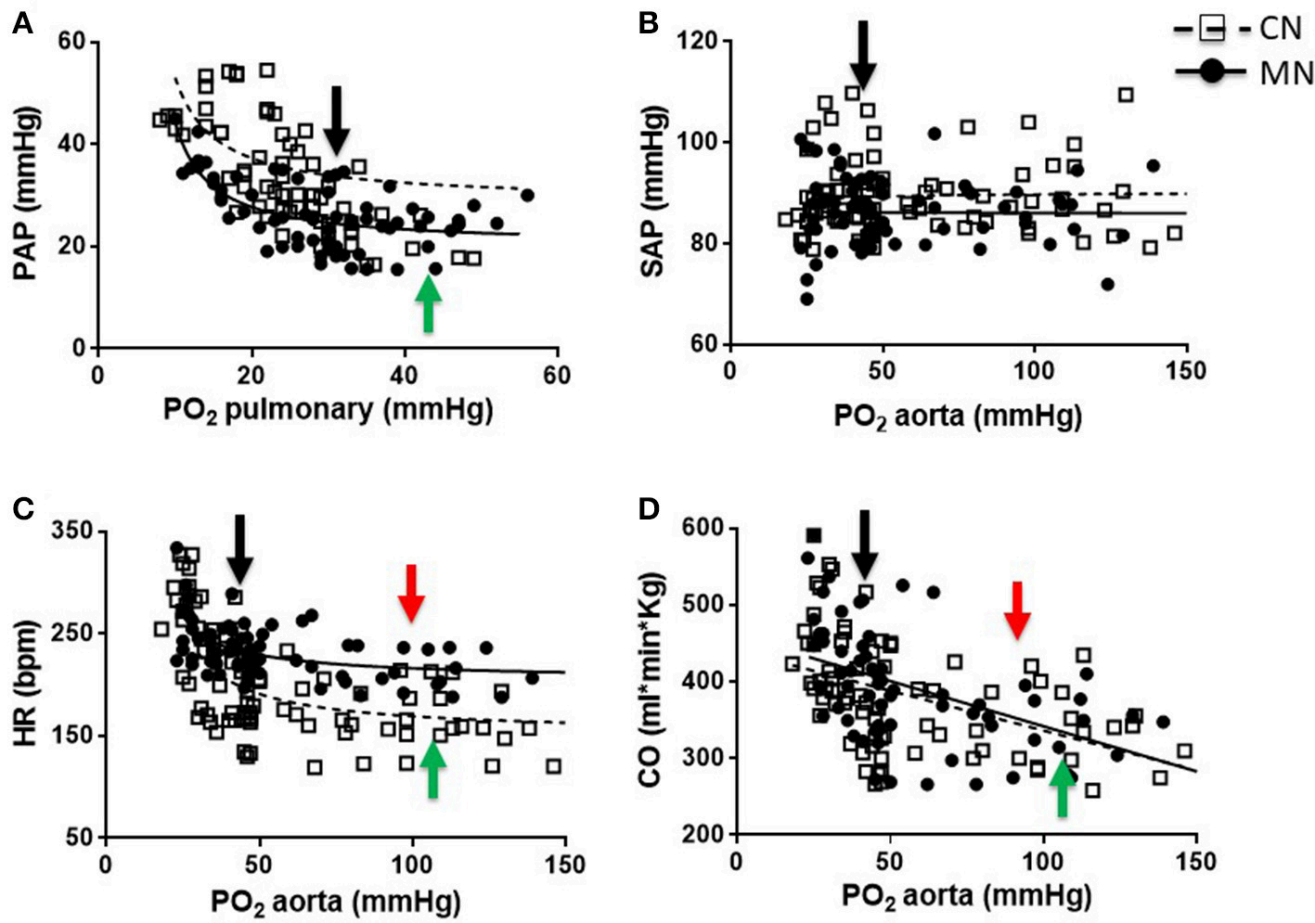
Cardiopulmonary responses to graded oxygenation. Correlations of PO_2_ vs. PAP **(A)**, PAS **(B)**, HR **(C)**, and CO **(D)** during the graded oxygenation protocol. Groups are control (CN, open squares, *n* = 6) and melatonin treated (MN, closed circles, *n* = 6) lambs. Values are means ± SEM. Lines represent the non-linear fit for CN (dashed line) and MN (continuous line) animals. Black arrows indicate baseline point at high altitude (breathing room air). Green and red arrows indicate inflection points for CN and MN animals, respectively.

The PAP values were similar at baseline between animals and both groups showed an inverse relation between PAP and PO_2_ during the graded oxygenation studies. However, the response was markedly blunted in the MN group relative to control neonates (Figure 1A). In fact, the slope of the curve in the MN group (−0.907 ± 0.078) was decreased relative to the control group (−2.114 ± 0.721). Additionally, MN neonates showed an inflection point on their PAP response (43.06 ± 2.48 mmHg PO_2_), not observed in control neonates. In marked contrast, SAP did not change with PO_2_ modifications and the values were similar between experimental groups (Figure 1B). Furthermore, basal HR was markedly increased in MN relative to CN group, but maintain a similar response pattern in which a decrease in PO_2_ determines an increase in HR (Figure 1C). However, the response intensity (slope) was lower in MN neonates (−0.984 ± 0.337 bpm/mmHg) compared to their controls (−2.394 ± 0.384 bpm/mmHg). Further, there were similar differences at high PO_2_ levels, where MN neonates showed a diminished chronotropic response (CN, 1.326 ± 0.343 vs. MN, 0.477 ± 0.008; *p* = 0.174). Control and MN neonates present inflection points at similar PO_2_ levels (CN, 98.35 ± 1.15 mmHg vs. MN, 107.20 ± 9.13 mmHg PO_2_; *p* = 0.310), but a higher HR is observed in MN neonates (CN, 147.90 ± 18.49 bpm vs. MN, 208.40 ± 13.44 bpm; *p* = 0.040). Moreover, CO was similar between groups and both tend to decrease as PO_2_ increase, at low levels of PO_2_, in both groups (CN, −2.814 ± 0.544 ml.min.kg/mmHg vs. MN, −3.185 ± 0.360 ml.min.kg/mmHg; *p* = 0.573) (Figure 1D). At higher PO_2_ levels, an inflection point is observed in both groups, but no significant differences were found in PO_2_ (CN, 94.62 ± 3.93 mmHg vs. MN, 105.60 ± 12.70 mmHg PO_2_; *p* = 0.433) or CO (CN, 296.40 ± 42.84 ml.min.kg vs. MN, 304.80 ± 19.53 ml.min.kg; *p* = 0.863).

### Histomorphometry and protein expression of pulmonary resistance arteries

**Figure 2:**
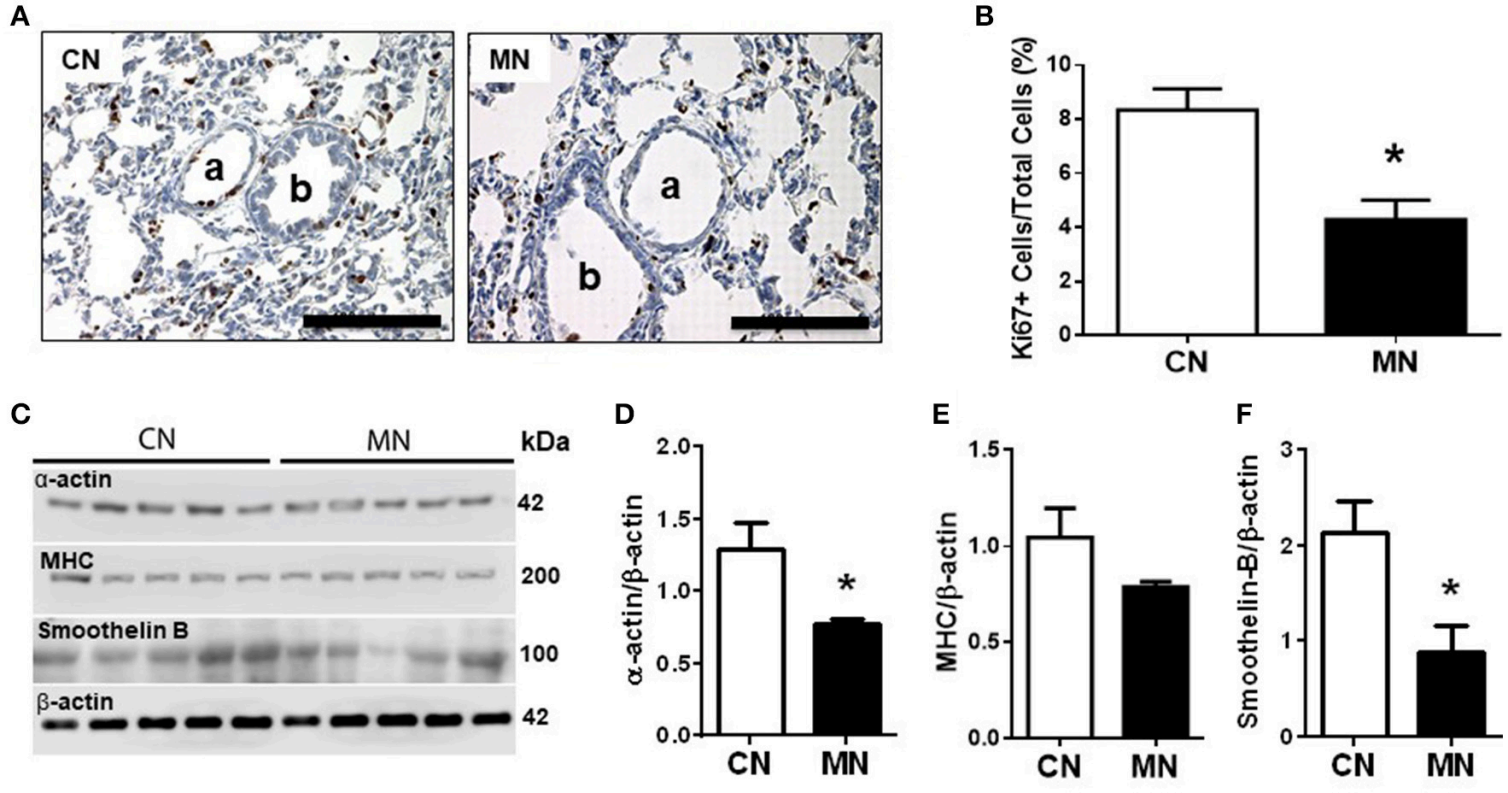
Pulmonary vascular remodeling. Representative micrographs (40x) of pulmonary tissue showing immunohistochemical distribution of Ki67 on pulmonary arteries **(A)**, and vascular Ki67+ cells/total cells quantification **(B)**. **a**: arteriole; **b**: bronchiole; scale bar: 100 μm. Scanned photograph of the Western blots **(C)**; Analyses for α-actin **(D)**, MHC **(E)**, and smoothelin-B **(F)** protein expression. Protein expression was referred to β-actin as control protein. Groups are control (CN, open bars, *n* = 5) and melatonin treated (MN, closed bars, *n* = 5) lambs. Values are means ± SEM. Significant differences (*P* ≤ 0.05): ^*^vs. CN.

Histomorphometric analysis of small pulmonary arteries (50–200 μm diameter) showed similar internal and external vascular diameters; luminal, media and adventitia areas between groups. However, the luminal/wall area ratio was higher in the group of neonates treated with melatonin relative to the control group (Table 2). In contrast, the media cellular density was markedly decreased in the group of neonates treated with melatonin (Table 2). These characteristics were associated with a diminished expression of the proliferation marker Ki67 (Figures 2A,B) and the remodeling markers, α-actin and smoothelin-B (Figures 2C,D,F) in the melatonin treated group. In contrast, MHC expression was similar between groups (Figures 2C,E).

**Table 2:** Morphometry of small pulmonary arteries.

	**Control (CN)**	**Melatonin (MN)**
Internal diameter (μm)	89.10 ± 2.48	95.25 ± 2.18
External diameter (μm)	125.2 ± 2.6	129.7 ± 2.4
Vascular wall thickness (μm)	18.04 ± 0.63	17.21 ± 0.67
Media thickness (μm)	7.51 ± 0.26	7.65 ± 0.24
Adventitia thickness (μm)	9.36 ± 0.36	8.51 ± 0.38
Luminal area/Wall area ratio (%)	50.98 ± 1.13	54.66 ± 1.24^*^
Media cellular density (cells/μm^2^)^*^100	1.79 ± 0.08	1.27 ± 0.07^*^

*Internal diameter, external diameter, wall thickness, media thickness, adventitia thickness, luminal area/wall area ratio, and media cellular density. Groups are control (CN, n = 6) and melatonin treated (MN, n = 6) lambs. Values are means ± SEM. Significant differences (P ≤ 0.05)*:

* *vs. CN*.

**Figure 3:**
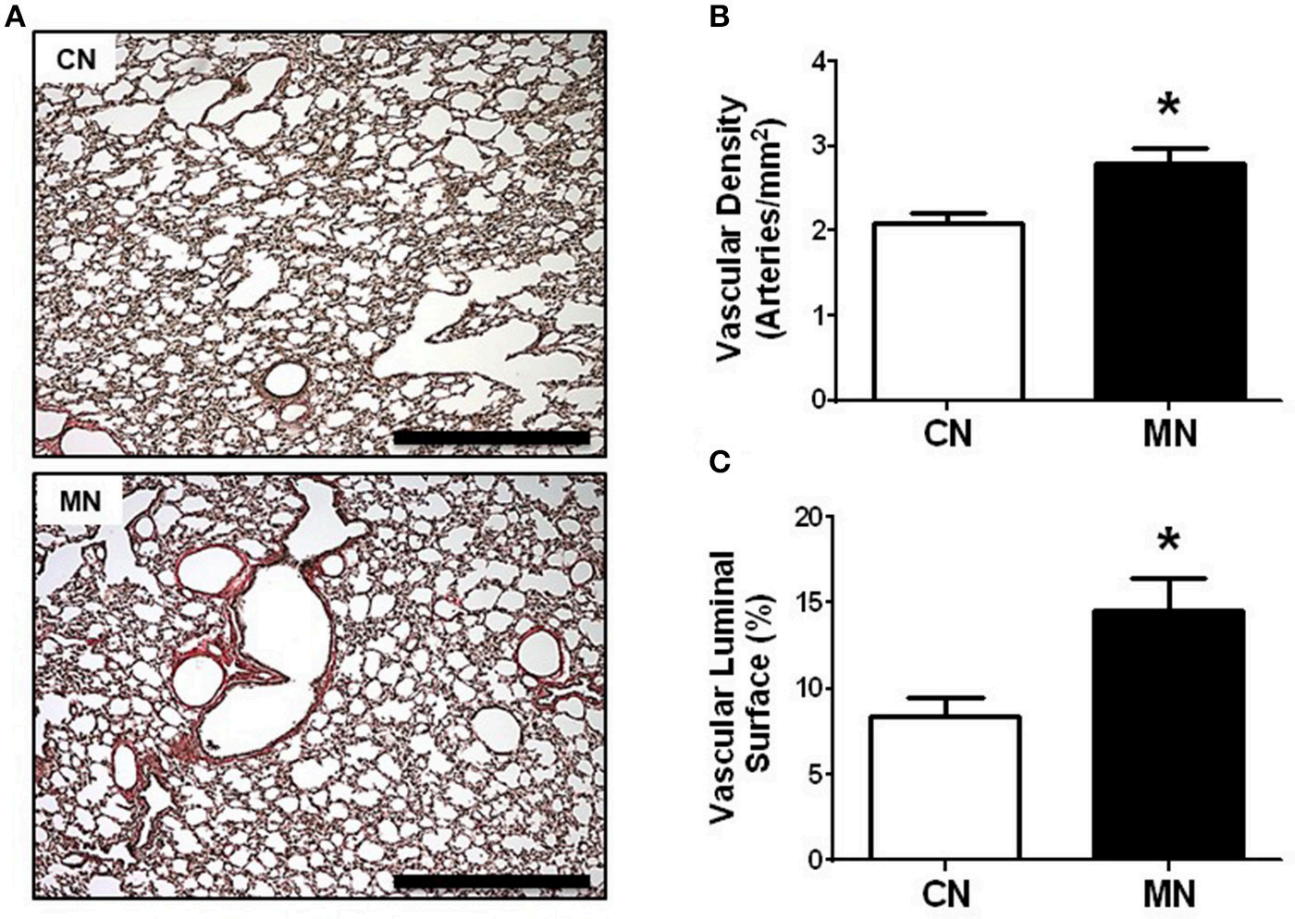
Vascular density and luminal surface area of pulmonary resistance arteries. Representative micrographs (10x) of Van Gieson stained lung sections **(A)**, vascular density **(B)**, and luminal surface area **(C)**. Groups are control (CN, open bars, *n* = 5) and melatonin treated (MN, closed bars, *n* = 5) lambs. Values are means ± SEM. Significant differences (*P* ≤ 0.05): ^*^vs. CN. Scale bar: 500 μm.

In addition, the analysis of vascular surface of the lung revealed a greater number of resistance arteries (50–200 μm) per area in the melatonin group relative to the control group (vascular density: CN, 2.08 ± 0.11 vs. MN, 2.79 ± 0.18 arteries/mm^2^; Figures 3A,B). Similarly, the vascular luminal surface expressed as percentage of lung parenchyma was higher in the melatonin treated group compared to the control group (CN, 8.43 ± 1.10 vs. MN, 14.56 ± 1.87%; Figures 3A,C).

**Figure 4:**
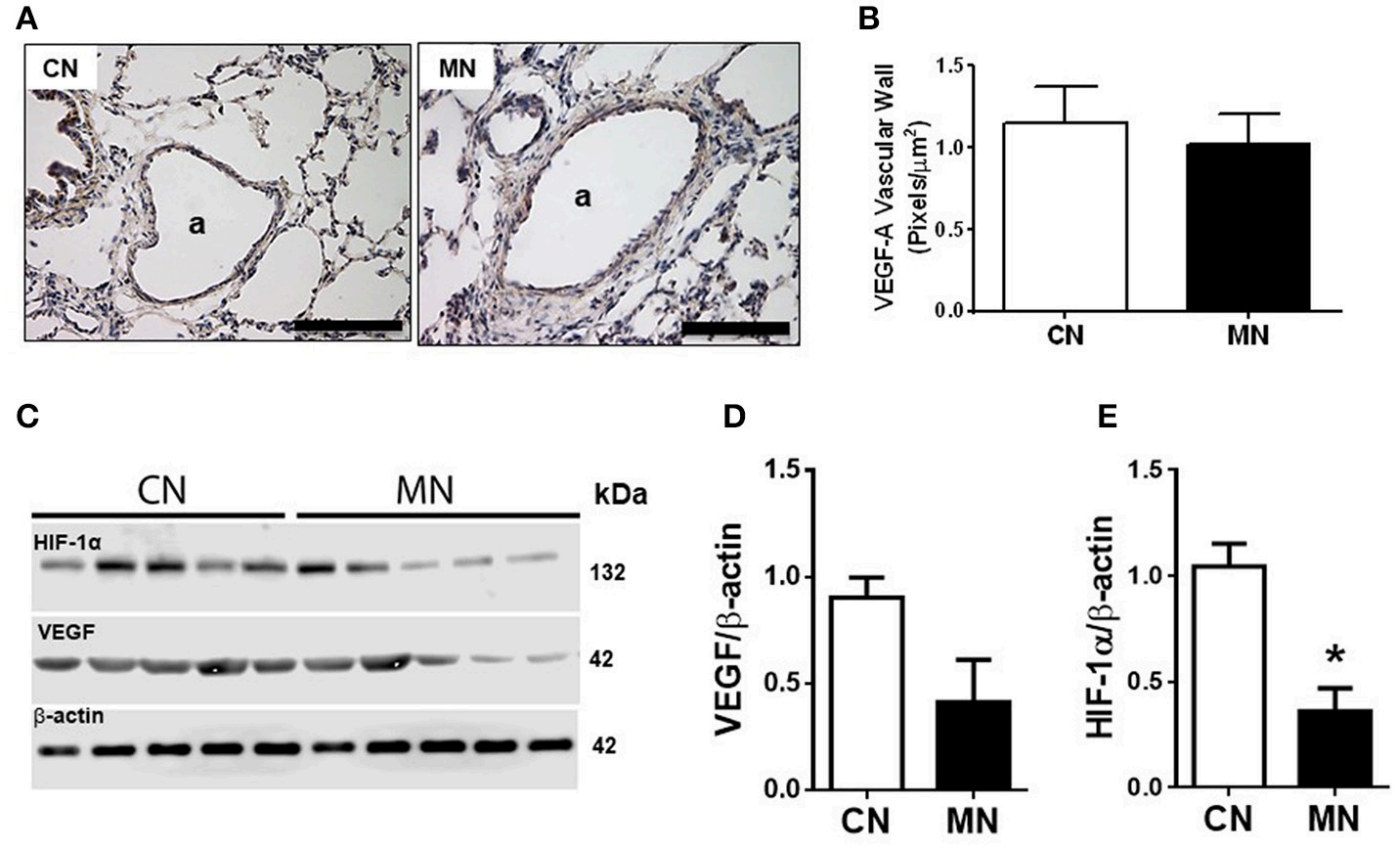
Immunolocalization and protein expression of VEGF and HIF in lung tissue. Representative micrographs (40x) showing immunohistochemical distribution of VEGF-A in vascular wall of lung resistance arteries **(A)**, and analysis for VEGF-A vascular immunoreactivity intensity **(B)**. Scale bar: 100 μm. Scanned photograph of immunoblots **(C)**, and HIF-1α **(D)** and VEGF **(E)** protein expression. Protein expression was referred to β-actin as control protein. Groups are control (CN, open bars, *n* = 5) and melatonin treated (MN, closed bars, *n* = 5) lambs. Values are means ± SEM. Significant differences (*P* ≤ 0.05): ^*^vs. CN.

Melatonin treatment did not change the immunoreactivity of pulmonary arteries for -VEGF-A (CN, 1.14 ± 0.22 vs. MN, 1.02 ± 0.17 pixels/μm^2^; Figures 4A,B) nor the total lung VEGF-A expression (CN, 0.903 ± 0.095 vs. MN, 0.413 ± 0.198; Figures 4C,D). Nevertheless, melatonin treatment induced a marked fall in HIF-1α in pulmonary tissue (CN, 1.045 ± 0.107 vs. MN, 0.361 ± 0.110; Figures 4C,E).

### Histomorphometry and protein expression of main pulmonary artery

**Figure 5:**
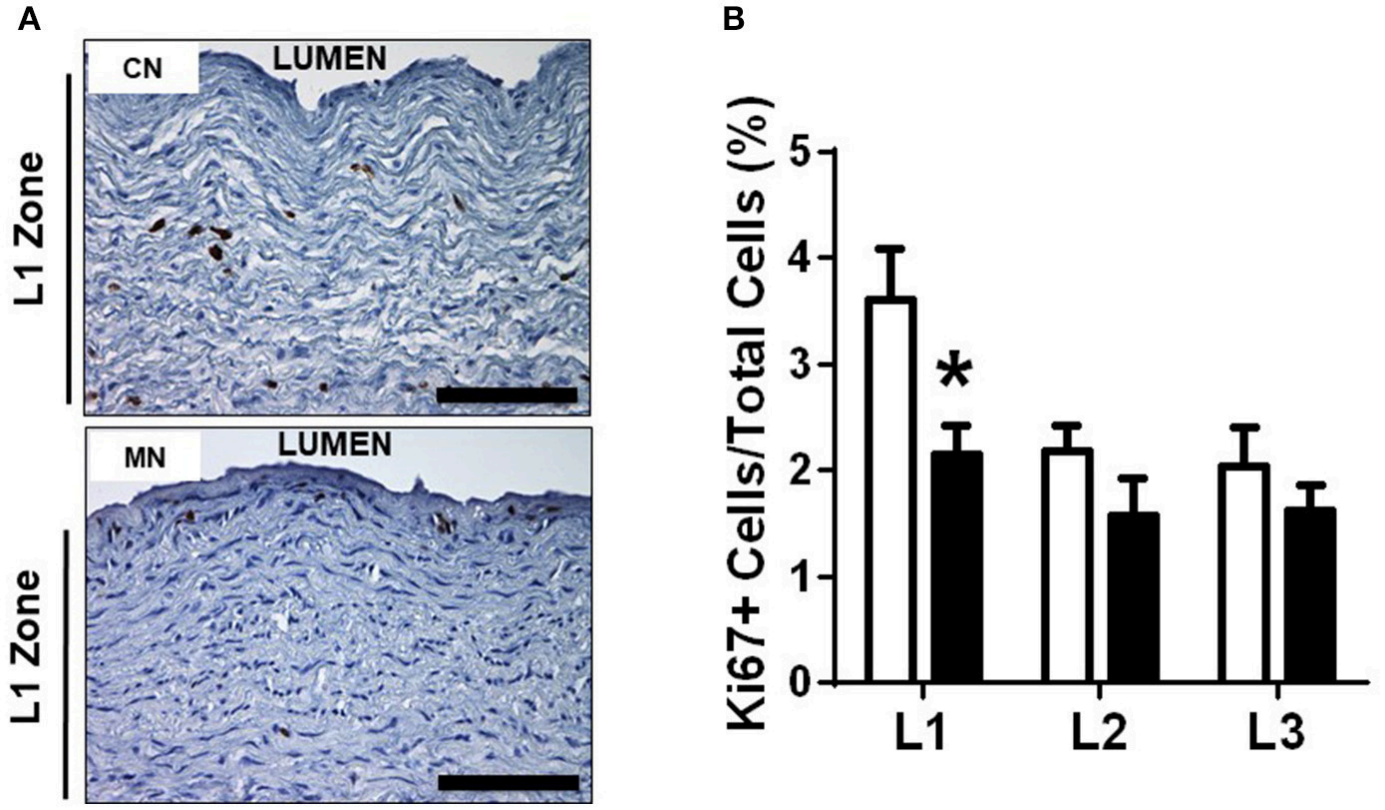
Pulmonary trunk remodeling. Representative micrographs (40x) of L1 zone of pulmonary trunk showing immunohistochemical distribution of Ki67+ **(A)** and analysis of % positive cells in L1, L2, and L3 zones of the pulmonary trunk **(B)**. Groups are control (CN, open bars, *n* = 6) and melatonin treated (MN, closed bars, *n* = 6) lambs. Values are means ± SEM. Significant differences (*P* ≤ 0.05): ^*^vs. CN. Scale bar: 100 μm.

Melatonin treatment increased the luminal diameter of the pulmonary trunk, without modifying the external diameter. Consequently, the tunica media showed a decreased thickness in the neonates treated with melatonin compared to the control group (Table 3). Similarly, we found a specific reduction in the L1 zone of the melatonin treated group, but with conserved thickness of L2 and L3 zones between groups (Table 3). The latter was associated with a reduced percentage of Ki-67 positive cells in the melatonin treated group compared to the control group, exclusively for the L1 zone (CN, 3.60 ± 0.47 vs. MN, 2.15 ± 0.26%Ki-67+ cells; Figure 5). In contrast, no differences were observed in L2 (CN, 2.17 ± 0.24 vs. MN, 1.57 ± 0.34%Ki-67+ cells) and L3 (CN, 2.04 ± 0.35 vs. MN, 1.61 ± 0.23%Ki-67+ cells) zones (Figure 5).

**Table 3:** Morphometry of main pulmonary artery.

	**Control (CN)**	**Melatonin (MN)**
Internal diameter (mm)	7.93 ± 0.53	9.85 ± 0.47^*^
External diameter (mm)	9.38 ± 0.74	11.02 ± 0.49
Media thickness (mm)	1.223 ± 0.037	1.119 ± 0.025^*^
L1 zone thickness (μm)	275.1 ± 10.4	223.0 ± 13.7^*^
L2 zone thickness (μm)	386.6 ± 15.7	391.6 ± 25.5
L3 zone thickness (μm)	546.7 ± 26.6	537.1 ± 37.4
L1 zone cellular density (cells/mm^2^)	5766 ± 112	5117 ± 219^*^
L2 zone cellular density (cells/mm^2^)	3671 ± 157	3404 ± 125
L3 zone cellular density (cells/mm^2^)	7336 ± 610	6981 ± 797

*Internal diameter, external diameter, media thickness, and L1, L2, L3 thicknesses and cellular density. Groups are control (CN, n = 6) and melatonin treated (MN, n = 6) lambs. Values are means ± SEM. Significant differences (P ≤ 0.05)*:

* *vs. CN*.

### Nitrotyrosine

**Figure 6:**
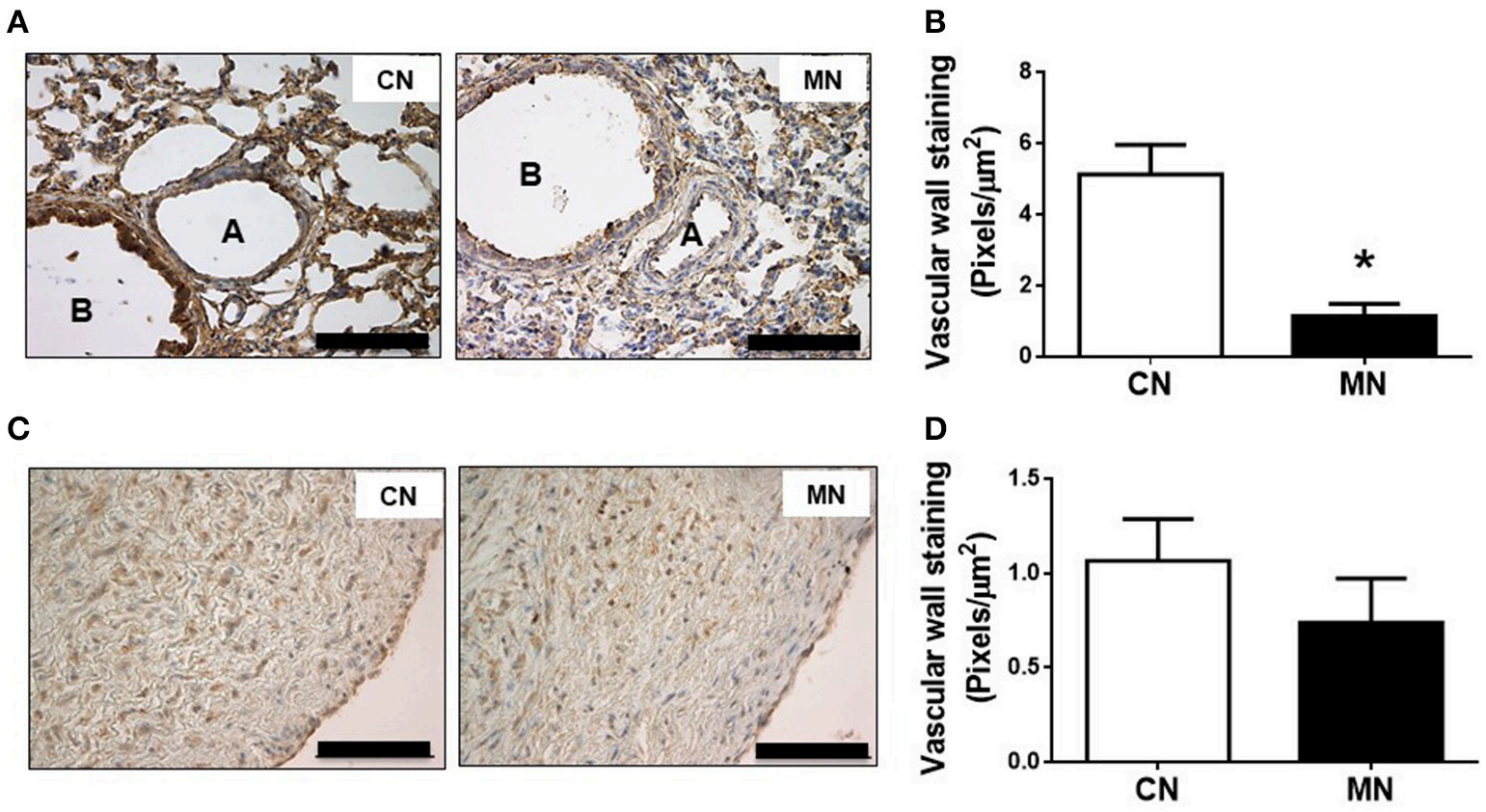
Pulmonary nitrosative stress. Representative micrographs (40x) of pulmonary resistance arteries **(A)** and nitrotyrosine intensity in pulmonary resistance arteries **(B)**. Representative micrographs (40x) of pulmonary trunk **(C)** and nitrotyrosine intensity in pulmonary trunk **(D)**. Groups are control (CN, open bars, *n* = 6) and melatonin treated (MN, closed bars, *n* = 6) lambs. Values are means ± SEM. Significant differences (*P* ≤ 0.05): ^*^vs. CN. Scale bar: 100 μm.

The immunoreactivity against nitrotyrosine, an oxidative stress marker, was lower in the small pulmonary arteries of MN group compared to the control group (CN, 5.12 ± 0.84 vs. MN, 1.14 ± 0.34 pixels/μm^2^; Figures 6A,B). In contrast, the immunoreactivity for nitrotyrosine in pulmonary trunk was similar in both groups (CN, 1.06 ± 0.22 vs. MN, 0.73 ± 0.23 pixels/μm^2^; Figures 6C,D).

## Discussion

Hypobaric hypoxia during gestation and early post-natal period impairs the physiological pulmonary vascular transition in the neonate (Papamatheakis et al., 2013; Lakshminrusimha et al., 2016). This impairment results in maladaptive changes of the function and structure of the pulmonary arteries that are characteristic of PHN. The currently approved treatments for this syndrome have limited effectiveness, maybe due to the modest anti-oxidant and anti-remodeling effects (Lakshminrusimha et al., 2016). Here, we further demonstrate that a postnatal treatment with melatonin improved function and decreased remodeling processes in PHN, reversing the pathological vascular phenotype. In fact, melatonin treatment diminishes exclusively the pulmonary vascular response to a hypoxic/hyperoxic challenge, through a decrease in oxygen-induced vasoactive responses. Further, melatonin promotes the vascular lung development, evidenced by increase vascular density and luminal surface area of the pulmonary arteries. In addition, melatonin increases significantly the luminal/vascular areas ratio and decreases vascular smooth muscle and remodeling markers in small pulmonary arteries. At the same time, several effects of the postnatal melatonin treatment are also observed in the pulmonary trunk so it seems that melatonin has a global effect in the pulmonary circulation, improving function and decreasing pathological vascular remodeling and oxidative stress. Previously, we have shown that melatonin could be beneficial as treatment for PHN acting as an antioxidant and improving the vasodilator capacity (Torres et al., 2015). All of the above findings support the idea that melatonin has anti-remodeling, antioxidant and vasodilator effects in PHN.

We have developed a test to assess cardiopulmonary responses to FiO_2_ modifications, which enable us to determine *in vivo* oxygen sensitivity (Herrera et al., 2007). As shown previously, chronic hypoxia determines an increased PVR and pressure at any PO_2_ level, relative to lowlanders. Therefore, a blunted vasoconstrictor response to acute hypoxia, shown in the present study, is reversing the high-altitude phenotype and balancing the vasoactive tone toward vasodilation when challenged. This could be explained by 2 facts: first, the antiremodelling and functional effects that decreases vasoconstriction and second, by the decreased oxidative stress that favors vasodilation due to decreased iCa^+2^ influx (Sylvester et al., 2012; Herrera et al., 2015).

Gestation and early postnatal life under hypobaric conditions induces remodeling in resistance arteries of the lung and it is characterized by a thicker media and a decreased luminal area relative to neonates conceived in lowlands (Haworth and Hislop, 1982; Stenmark et al., 1987, 1999; Herrera et al., 2008, 2010; Papamatheakis et al., 2013). Even though postnatal melatonin treatment did not alter significantly the morphological variables studied in small pulmonary arteries, a mild increase in luminal area and a slight decrease in the vascular area, increase significantly the ratio between luminal and vascular areas. This is a remarkable finding because decreased luminal area vs. vascular area is, in fact, the main hallmark of the remodeling of resistance arteries (Stenmark et al., 1987; Mulvany, 1999). At the same time, resistance arteries remodeling is associated with SMCs hyperplasia in pulmonary hypertension and oxidative stress (Jaitovich and Jourd'heuil, 2017). Interestingly, here we demonstrated that melatonin treatment also decreases media cellular density and SMCs proliferation, showing a potential anti-proliferative effect. Similar results have been described using an adult rat model with pulmonary hypertension treated with melatonin (Hung et al., 2013; Jin et al., 2014). In this regard, we further demonstrate that melatonin also decreases α-actin and smoothelin-B pulmonary expression, which are SMC markers. Taken together, these findings support the idea that melatonin modulates the SMCs cell cycle and proliferation.

Perinatal hypoxia impairs fetal to neonatal lung morphofunctional transition determining pulmonary underdevelopment in some cases and increased lung maturation in other reports (Braems et al., 2000; Orgeig et al., 2010; McGillick et al., 2016, 2017). The different outcomes may be explained by different animal models, and/or timing and intensity of the hypoxic insult (McGillick et al., 2017). Our findings demonstrate that melatonin treatment stimulates vascular lung development, increasing vascular density and luminal surface of pulmonary arteries in PHN. However, we did not observe VEGF increases mediating an enhanced vascularization. Nevertheless, we cannot rule-out changes in VEGF-signaling, at a receptor or at a post-receptor level. We propose that the decreased activation of HIF-1 is modulating this lack of VEGF increase. As ROS stabilizes HIF-1α expression (Wheaton and Chandel, 2011; Prabahakar and Semenza, 2012), the antioxidant effects of melatonin may reduce HIF-1α. This is consistent with our results of nitrotyrosine drop in pulmonary resistance arteries. Although we did not measure HIF-1α in nuclear extracts to indicate activation, a lower protein content in parenchyma strongly suggests an increased degradation of HIF. Further, HIF signaling seems to be also triggered by vascular stretch in neonates, by mechanisms involving mitochondrial ROS and NFκB (Wedgwood et al., 2015). Therefore, a decreased stretching stimuli, for instance in a more flexible or plastic circulation, is another way by which HIF is reduced, consistent with the diminished vasoactive response to an acute hypoxic challenge.

This same effect has been shown in adult rats (Jin et al., 2014) and may be another important effect of melatonin as HIF-driven pathophysiological processes are involved in hypoxia-induced remodeling (Veith et al., 2016). Interestingly, a recent study in hypoxic adult rats with pulmonary hypertension found that the right ventricular pressure and the pulmonary arteriolar wall thickness were significantly increased in hypoxic animals with elevated levels of malondialdehyde. These effects were significantly attenuated with a melatonin treatment (Hung et al., 2017).

In our study, the vascular anti-remodeling effect was present in the intrapulmonary arteries and the pulmonary trunk, probably attenuating the remodeling process of the entire pulmonary circulation. However, melatonin decreases the total medial thickness in the pulmonary trunk through a specific effect in L1 zone, decreasing SMCs cellular density and proliferation. This finding support the idea that different compartments exhibit specific growth capacities and responses (Frid et al., 1997b), and that L1 zone might be the responsible for the chronic hypoxia-induced remodeling. In addition, this layer may have a higher expression of melatonin (MT) receptors, explaining the specific response. However, to the best of our knowledge, there are no studies about the distribution of MT receptors on the pulmonary trunk. In fact, only a few studies showed that porcine and rodents seem to have functional MT receptors in the pulmonary artery but without expression studies (Ting et al., 2000; Drew et al., 2001; Das et al., 2008).

One of the limitations of our study is that we do not know if the effects induced by melatonin are receptor-mediated. Previous studies have shown that MT1 and MT2 receptors are present in lung from adult animals (Naji et al., 2004) and stressed babies (Olegário et al., 2013). These studies and others (Torres et al., 2015) have proposed that some of the vasodilator effects of melatonin are receptor-mediated and some are not. All of the findings in our study imply a vascular regulation of melatonin, unfortunately it still remain unknown whether these effects are via a receptor dependent or independent mechanism.

We administered melatonin after sunset to follow and enhance the physiological increase of this neurohormone during night. In a previous study we reported that the plasma levels of melatonin increased ~5-fold during night after a similar administration regimen in newborns (Torres et al., 2015). Furthermore, melatonin concentration at the next morning was similar in treated and control sheep (Torres et al., 2015; González-Candia et al., 2016), evidencing two important facts: melatonin has a short half-life (Harpsøe et al., 2015) and that the findings of this study are induced by an increase in the plasma melatonin concentration during night time. Further, we did not observe any dizziness of behavioral alteration in melatonin treated neonates relative to controls. In fact, apparently the have the same physical activity and kept the daily gain weight (Torres et al., 2015).

Our findings add robust knowledge to the evidence found in previous studies (Hung et al., 2013, 2017; Jin et al., 2014; Torres et al., 2015) suggesting that melatonin treatment has a protective effect on pulmonary hypertension induced by chronic hypoxia through vascular functional and anti-remodeling effects.

## Conclusions

Although current treatments such as iNO therapy has successfully improved clinical management of PHN, more studies are still needed to advance in the effectiveness of the PHN therapeutical approach (Jain and McNamara, 2015). We suggest that the administration of antioxidants and anti-remodeling agents associated with the current vasodilator therapies might improve the treatment outcome. From this point of view, melatonin appears as an effective agent for PHN, markedly improving pulmonary vascular function (Torres et al., 2015). Future studies should focus on optimizing doses and/or therapeutic windows to boost the functional and anti-remodeling effects described in this study. At the moment, our findings strongly suggest that melatonin might be an effective adjuvant for the currently approved therapies for PHN by promoting beneficial functional and structural changes in the pulmonary circulation.

## Author contributions

CA, AG-C, GE, RR, AL, and EH: conceived and designed the experiments; CA, AG-C, AC, EF, DC, and EH: collected and analyzed the data; CA, AG-C, AC, EF, DC, GE, RR, AL, and EH: interpreted and discussed the outcomes; CA, AG-C, and EH: drafted the article. All authors revised it critically and approved the final version.

### Conflict of interest statement

The authors declare that the research was conducted in the absence of any commercial or financial relationships that could be construed as a potential conflict of interest. The reviewer VGD and handling Editor declared their shared affiliation.
